# Seasonal Dynamics of Malaria in Pregnancy in West Africa: Evidence for Carriage of Infections Acquired Before Pregnancy Until First Contact with Antenatal Care

**DOI:** 10.4269/ajtmh.17-0620

**Published:** 2017-12-04

**Authors:** Isha Berry, Patrick Walker, Harry Tagbor, Kalifa Bojang, Sheick Oumar Coulibaly, Kassoum Kayentao, John Williams, Abraham Oduro, Paul Milligan, Daniel Chandramohan, Brian Greenwood, Matthew Cairns

**Affiliations:** 1 London School of Hygiene and Tropical Medicine, London, United Kingdom;; 2 Imperial College London, London, United Kindom;; 3 University of Health and Allied Sciences, Ho, Ghana;; 4 Medical Research Council Unit, Fajara, The Gambia;; 5 World Health Organization, Regional Office for Africa, Brazzaville, Congo;; 6 Malaria Research and Training Center, Faculty of Medicine and Odontostomatology, University of Sciences, Techniques and Technologies, Bamako, Mali;; 7 Navrongo Health Research Center, Navrongo, Ghana

## Abstract

In malaria-endemic areas, *Plasmodium falciparum* prevalence is often high in young women because of 1) low use of insecticide-treated nets before their first pregnancy and 2) acquired immunity, meaning infections are asymptomatic and thus untreated. Consequently, a common source of malaria in pregnancy (MiP) may be infected women becoming pregnant, rather than pregnant women becoming infected. In this study, prevalence of infection was determined by microscopy at first antenatal care (ANC) visit in primigravidae and secundigravidae in Ghana, Burkina Faso, Mali, and The Gambia, four countries with strong seasonal variations in transmission. Duration of pregnancy spent in the rainy season and other risk factors for infection were evaluated using multivariable Poisson regression. We found that the overall prevalence of malaria at first ANC was generally high and increased with time spent pregnant during the rainy season: prevalence among those with the longest exposure was 59.7% in Ghana, 56.7% in Burkina Faso, 42.2% in Mali, and 16.8% in Gambia. However, the prevalence was substantial even among women whose entire pregnancy before first ANC had occurred in the dry season: 41.3%, 34.4%, 11.5%, and 7.8%, respectively, in the four countries. In multivariable analysis, risk of infection was also higher among primigravidae, younger women, and those of lower socioeconomic status, independent of seasonality. High prevalence among women without exposure to high transmission during their pregnancy suggests that part of the MiP burden results from long-duration infections, including those acquired preconception. Prevention of malaria before pregnancy is needed to reduce the MiP burden.

## INTRODUCTION

Malaria in pregnancy (MiP) is a major public health concern, particularly within sub-Saharan Africa, which shoulders a disproportionately large share of the malaria burden.^1–3^ Within this region, up to 9.5 million pregnant women were estimated to be at risk of *Plasmodium falciparum* infection in 2015.^4^ Infection during pregnancy is not only deleterious to the woman but it also puts her fetus at increased risk of adverse outcomes, such as preterm delivery, low birth weight, and intrauterine growth restriction.^5–8^ Recommended strategies to prevent and treat MiP include free distribution of nets through antenatal care (ANC) and intermittent preventive treatment (IPTp), which involves providing a dose of sulfadoxine–pyrimethamine (SP) to pregnant women at each ANC visit during the second and third trimesters of their pregnancy.^9^ However, neither of these interventions takes place until a woman reaches ANC, which can leave her unprotected for almost the first half of her pregnancy. Given the burden of MiP, which in some countries has persisted despite the scaling-up of IPTp and delivery of nets, there remains a need to identify women at greatest risk to inform additional interventions.^9,10^

It has recently been suggested that a large percentage of malaria infections carried in early pregnancy reflect infections acquired before conception (i.e., the order of events being infected women become pregnant, rather than pregnant women become infected).^11^ Evidence from such models are supported by a number of well-known features of malaria epidemiology in high transmission areas, including a very high prevalence of malaria infection often observed at first ANC visit,^12^ high prevalence of asymptomatic (and thus untreated) infections in adolescents and young women (i.e., the source population for pregnancies),^13,14^ and low bed net use among young women—particularly before first pregnancy when nets are distributed through ANC.^13,15^

Identifying whether infections observed during pregnancy result from infections acquired before pregnancy, or since conception, may have important implications for malaria control.^16^ If infections acquired preconception are an important source of MiP, then it will be necessary to target nonpregnant women to reduce the risk of MiP, and its associated burden, in these women. However, estimating when malaria infections were acquired, and their duration, is difficult in practice. Areas of highly seasonal malaria transmission, such as the Sahel region of sub-Saharan Africa, where infection risk is largely confined to a few months each year, provide an opportunity to examine this issue.^17^ In such settings, there is a well-established association between seasonality and malaria risk,^18–20^ and the timing of the malaria season is well defined.^20,21^ Therefore, depending on the time of conception and the time of first ANC contact, it can be inferred with a reasonable degree of certainty whether women have spent all, some, or none of their pregnancy exposed to high levels of malaria transmission. Although transmission of malaria does not entirely stop during the dry season, if prevalence at the first ANC visit is still high among women who have been exposed only to low levels of transmission since they became pregnant, this could suggest that infections are acquired preconception (during the peak season). Such infections are likely to have been carried until first presentation at an ANC, which is usually a woman’s first opportunity to receive a curative course of IPTp.

In this study, we estimate the prevalence of MiP in four areas of highly seasonal malaria transmission in West Africa (Burkina Faso, The Gambia, Ghana, and Mali). We investigate risk factors for infection and evidence for carriage of infections acquired before pregnancy until the time of first ANC contact. The implications our findings may have on when, and for whom, additional MiP interventions could be introduced are discussed.

## METHODS

### Data collection and study sites.

**Figure 1.**
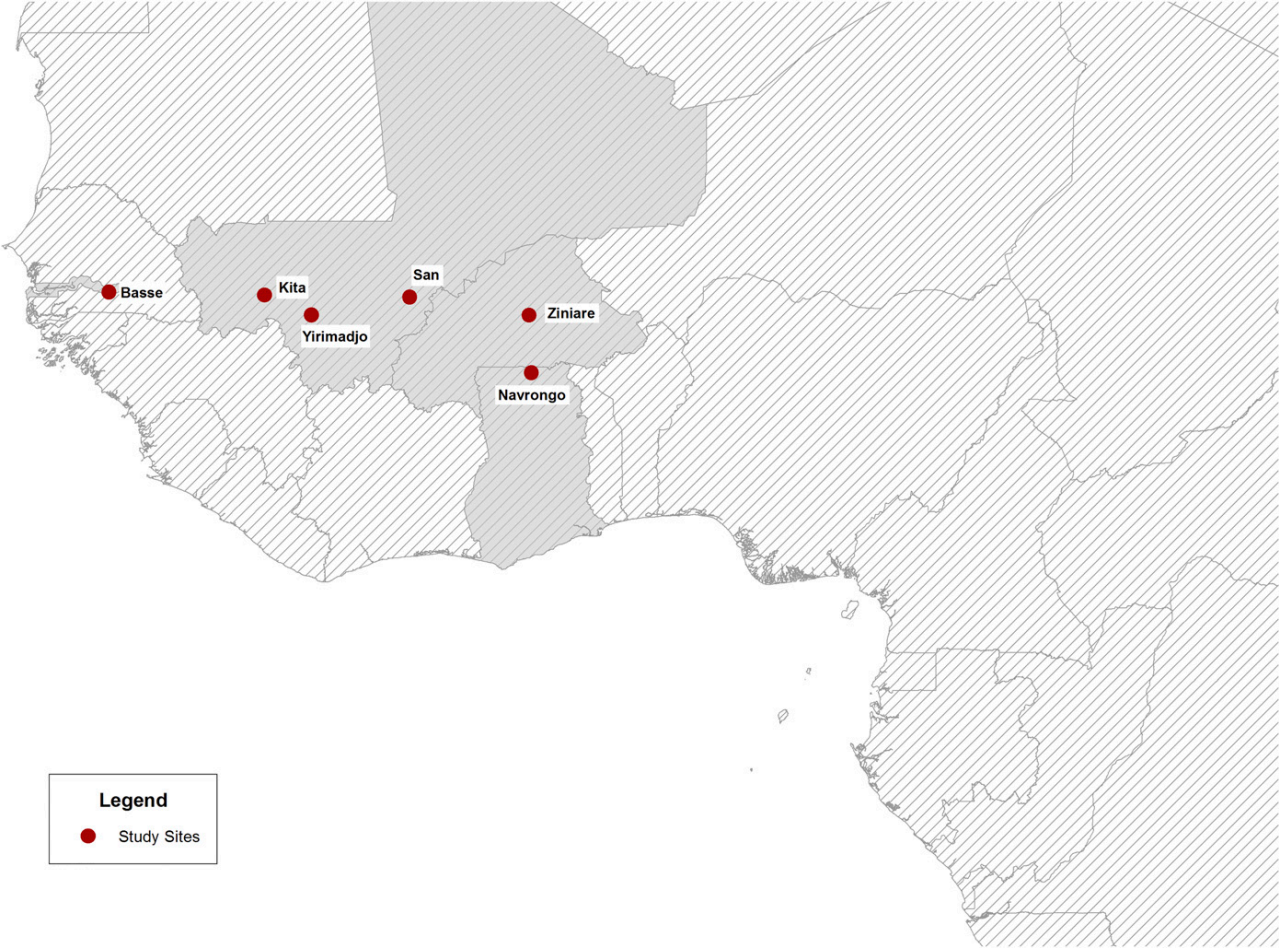
Map of West Africa highlighting locations of study centers. This figure appears in color at www.ajtmh.org.

This study uses data collected from a multicenter, individually randomized, noninferiority trial conducted in Burkina Faso, The Gambia, Ghana, and Mali. Details of the methods used in the trial have been presented previously.^12^ In brief, 5,354 primigravid or secundigravid women who attended their first ANC visit between May 2010 and October 2011 were randomized to receive either IPTp or intermittent screening and treatment (ISTp) on up to three occasions during their pregnancy. Women were followed until approximately 6 weeks postpartum, and a number of pregnancy outcomes were recorded. The study sites were Navrongo, Ghana; Ziniaré, Burkina Faso; Kita, Yirimadjo and San, Mali; and Bassé, The Gambia (Figure 1). The duration and timing of the wet and dry seasons vary between these sites, with corresponding differences in the length of the malaria high transmission season. Typically, malaria transmission peaks between June and November (i.e., 6 months) in Navrongo, between July and November (i.e., 5 months) in Burkina Faso and Mali, and between August and November (i.e., 3–4 months) in The Gambia. Although there is some year-to-year variation in the onset of the rainy season each year, there were no major departures from the typical seasonal pattern reported in any center over the study period. Transmission is relatively low at each site during the rest of the year.^22^ For instance, in Navrongo, the site with the longest season, transmission as measured by the entomological inoculation rate (EIR) has been shown to fall to very close to zero during the dry season, from a peak of between four and six infectious bites per day during the rainy season.^23^

The present analysis uses data collected from women attending an ANC clinic for the first time between 16 and 30 weeks of gestation. At this baseline visit, biological, social, and demographic characteristics were recorded, and a blood sample was obtained. Blood films were examined for malaria parasites by two independent readers, with discrepancies resolved using standardized methods.^24^ Nonfalciparum malaria is very rare in these countries.^25^ The blood film was defined as positive if any form of *P. falciparum* was observed, otherwise it was considered negative.

### Data analysis.

For each woman, the month of conception was estimated based on the date of the first ANC visit and the woman’s estimated gestational age, as measured by symphysis-fundal height.^12^ The duration of pregnancy occurring in the rainy season before the first ANC visit was calculated to estimate the number of months of high malaria risk to which each woman had been exposed, based on the seasonal patterns described above (see Supplemental Figure 1). Consequently, the number of months of pregnancy exposed to high transmission ranged from 0 to 6 months in Ghana, from 0 to 5 months in Burkina Faso and Mali, and from 0 to 4 months in The Gambia. Data on participants’ durable household assets and amenities (see Supplemental Text 1) were used to create socioeconomic status (SES) quintiles separately within each country using principal components analysis.^12^

In each country, sociodemographic and biological risk factors for *P. falciparum* infection were investigated, as well as the change in *P. falciparum* prevalence by month and by duration of pregnancy in the rainy season. Crude prevalence ratios (PRs) were calculated for malaria infection by each risk factor using Poisson regression with robust standard errors, as described previously.^26^ Multivariable models were then built for each country to obtain adjusted PRs with 95% confidence intervals (CIs), adjusted for potential confounding from other risk factors. We present results from all countries in the order of Ghana, Burkina Faso, Mali, and The Gambia, which correspond approximately to increasing seasonality and reducing transmission intensity. All analyses were conducted in Stata 13.0 (StataCorp, College Station, TX).

### Ethics.

The IPTp–ISTp trial received ethical approval from the committees of each of the participating African institutions (see Supplemental Text 2) as well as by the ethics committee of The London School of Hygiene and Tropical Medicine (LSHTM). The trial organizers obtained informed, written consent from all participants. The current study received separate ethical approval from The LSHTM Ethics Committee.

## RESULTS

### Population characteristics and prevalence.

Blood smear results were available at baseline for 5,237 women: 1,301 from Ghana, 1,434 from Burkina Faso, 1,308 from Mali, and 1,194 from The Gambia. In each country, those aged between 18 and 20 years accounted for the largest group. Primigravid women represented slightly over half of women enrolled, except in Burkina Faso where most of them were secundigravid. Bed net use at first ANC visit varied greatly between settings, with Ghanaian women reporting the lowest frequency of net use (37.4%) and Gambian women the highest (81.7%) (Tables 1–4). However, in all four countries, we found evidence of higher net use during second pregnancies (Ghana: primigravid [29.3%] versus secundigravid [47.6%]; Burkina Faso: 48.0% versus 59.8%; Mali 56.0% versus 69.7%; The Gambia: 82.3% versus 86.8%). Further analysis of net use by age and gravidity are shown in Supplemental Figure 2.

**Table 1:** Distribution of risk factors, crude and adjusted prevalence ratios for malaria in pregnancy in Ghana (crude *N* = 1,301; adjusted *N* = 1,293)

Variable	Frequency (%)	% *Plasmodium falciparum* infection	Crude PR* (95% CI)	*P* value†	Adjusted PR‡ (95% CI)	*P* value†
Months pregnant in rainy season				< 0.001		< 0.001
0 months	184 (14.1)	41.3	ref	–	ref	–
1 months	277 (21.3)	33.2	0.80 (0.63–1.02)	–	0.86 (0.68–1.08)	–
2 months	215 (16.5)	44.7	1.08 (0.86–1.36)	–	1.11 (0.89–1.39)	–
3 months	212 (16.3)	60.4	1.46 (1.19–1.79)	–	1.47 (1.21–1.78)	–
4 months	190 (14.6)	61.6	1.49 (1.21–1.83)	–	1.50 (1.23–1.82)	–
5 months	166 (12.8)	48.8	1.18 (0.94–1.49)	–	1.20 (0.96–1.50)	–
6 months	57 (4.4)	59.7	1.44 (1.10–1.90)	–	1.48 (1.13–1.95)	–
Age				< 0.001		< 0.001
25 years +	273 (21.0)	34.4	ref	–	ref	–
21–24 years	407 (31.3)	36.9	1.07 (0.87–1.32)	–	1.03 (0.84–1.27)	–
18–20 years	531 (40.8)	60.1	1.74 (1.46–2.08)	–	1.51 (1.25–1.84)	–
Under 18 years	90 (6.9)	67.8	1.97 (1.58–2.45)	–	1.55 (1.23–1.97)	–
Gravidity§				< 0.001		< 0.001
Secundigravid	575 (44.2)	37.7	ref	–	ref	–
Primigravid	723 (55.6)	56.0	1.48 (1.31–1.68)	–	1.29 (1.13–1.48)	–
Gestational age				0.08		0.02
< 20 weeks	567 (43.6)	47.8	ref	–	ref	–
20–24 weeks	602 (46.3)	50.2	1.05 (0.93–1.18)	–	1.01 (0.90–1.13)	–
25–30 weeks	132 (10.1)	38.6	0.81 (0.64–1.02)	–	0.74 (0.59–0.92)	–
Socio-economic status§				< 0.001		0.15
Least poor	262 (20.1)	37.0	ref	–	ref	–
Less poor	260 (20.0)	48.9	1.32 (1.08–1.61)	–	1.20 (0.98–1.45)	–
Middle	274 (21.1)	46.4	1.25 (1.02–1.53)	–	1.10 (0.90–1.34)	–
More poor	246 (18.9)	51.2	1.38 (1.13–1.69)	–	1.19 (0.98–1.44)	–
Most poor	255 (19.6)	56.5	1.53 (1.26–1.85)	–	1.26 (1.04–1.53)	–
Bed net use previous night§				0.05		0.01
Yes	487 (37.4)	51.5	ref	–	ref	–
No	813 (62.5)	45.9	0.89 (0.79–1.00)	–	0.85 (0.76–0.96)	–

CI = confidence interval; PR = prevalence ratio; ref = reference.

* Crude PRs calculated from univariate Poisson regression.

† *P* values obtained from Wald’s test. Comparisons restricted to observations without missing data for variables listed.

‡ Adjusted PRs calculated using Poisson regression, adjusting for all other variables listed in the table.

§ Missing values for gravidity *N* = 3 (0.2%); SES *N* = 4 (0.3%); bed net use *N* = 1 (0.1%).

**Table 2:** Distribution of risk factors, crude and adjusted prevalence ratios for malaria in pregnancy in Burkina Faso (crude *N* = 1,434; adjusted *N* = 1,330)

Variable	Frequency (%)	% *Plasmodium falciparum* infection	Crude PR* (95% CI)	*P* value†	Adjusted PR‡ (95% CI)	*P* value†
Months pregnant in rainy season§				< 0.001		< 0.001
0 months	262 (18.3)	34.4	ref	–	ref	–
1 months	287 (20.0)	27.9	0.81 (0.63–1.04)	–	0.87 (0.68–1.13)	–
2 months	272 (19.0)	43.8	1.27 (1.03–1.58)	–	1.38 (1.11–1.71)	–
3 months	207 (14.4)	44.0	1.28 (1.02–1.61)	–	1.34 (1.06–1.68)	–
4 months	205 (14.3)	53.7	1.56 (1.27–1.93)	–	1.70 (1.38–2.10)	–
5 months	180 (12.6)	56.7	1.65 (1.34–2.04)	–	1.85 (1.50–2.29)	–
Age				< 0.001		< 0.001
25 years+	131 (9.1)	23.7	ref	–	ref	–
21–24 years	433 (30.2)	29.1	1.23 (0.87–1.73)	–	1.25 (0.88–1.76)	–
18–20 years	855 (59.6)	51.0	2.15 (1.57–2.95)	–	1.81 (1.30–2.53)	–
Under 18 years	15 (1.1)	60.0	2.54 (1.51–4.24)	–	1.67 (0.73–3.82)	–
Gravidity§				< 0.001		< 0.001
Secundigravid	750 (52.3)	30.4	ref	–	ref	–
Primigravid	663 (46.2)	54.9	1.81 (1.59–2.05)	–	1.43 (1.24–1.65)	–
Gestational age§				0.003		0.002
< 20 weeks	489 (34.1)	47.0	ref	–	ref	–
20–24 weeks	750 (52.3)	40.8	0.87 (0.76–0.99)	–	0.85 (0.75–0.96)	–
25–30 weeks	174 (12.1)	32.2	0.68 (0.54–0.87)	–	0.69 (0.55–0.88)	–
Socio-economic status§				0.35		0.16
Least poor	280 (19.5)	41.8	ref	–	ref	–
Less poor	264 (18.4)	41.7	1.00 (0.82–1.22)	–	0.92 (0.76–1.11)	–
Middle	281 (19.6)	45.2	1.08 (0.90–1.31)	–	1.02 (0.85–1.21)	–
More poor	266 (18.6)	45.1	1.08 (0.89–1.31)	–	1.03 (0.86–1.24)	–
Most poor	270 (18.8)	37.4	0.90 (0.73–1.10)	–	0.83 (0.68–1.01)	–
Bed net use previous night§				0.06		0.10
Yes	769 (53.6)	39.9	ref	–	ref	–
No	651 (45.4)	44.9	1.12 (0.99–1.27)	–	1.11 (0.98–1.25)	–

CI = confidence interval; PR = prevalence ratio; ref = reference.

* Crude PRs calculated from univariate Poisson regression.

† *P* values obtained from Wald’s test. Comparisons restricted to observations without missing data for variables listed.

‡ Adjusted PRs calculated using Poisson regression, adjusting for all other variables listed in the table.

§ Missing values for season *N* = 21 (1.5%); gravidity *N* = 21 (1.5%); gestational age (1.5%); SES *N* = 73 (5.1%); bed net use *N* = 14 (1.0%).

**Table 3:** Distribution of risk factors, crude and adjusted prevalence ratios for malaria in pregnancy in Mali (crude *N* = 1,308; adjusted *N* = 1,258)

Variable	Frequency (%)	% *Plasmodium falciparum* infection	Crude PR* (95% CI)	*P* value†*	Adjusted PR‡ (95% CI)	*P* value†
Months pregnant in rainy season				< 0.001		< 0.001
0 months	339 (25.9)	11.5	ref	–	ref	–
1 months	283 (21.6)	14.5	1.26 (0.84–1.90)	–	1.19 (0.79–1.79)	–
2 months	263 (20.1)	24.3	2.12 (1.47–3.04)	–	1.98 (1.37–2.87)	–
3 months	168 (12.8)	21.4	1.86 (1.23–2.82)	–	1.66 (1.09–2.51)	–
4 months	165 (12.6)	40.0	3.48 (2.45–4.93)	–	2.81 (1.97–4.01)	–
5 months	90 (6.9)	42.2	3.67 (2.51–5.38)	–	3.16 (2.12–4.70)	–
Age				0.002		0.004
25 years+	107 (8.2)	11.2	ref	–	ref	–
21–24 years	243 (18.6)	16.5	1.47 (0.80–2.68)	–	1.57 (0.87–2.84)	–
18–20 years	606 (46.3)	25.7	2.30 (1.32–3.98)	–	2.00 (1.16–3.45)	–
Under 18 years	352 (26.9)	21.6	1.93 (1.09–3.40)	–	1.42 (0.80–2.52)	–
Gravidity				< 0.001		< 0.001
Secundigravid	512 (39.1)	14.8	ref	–	ref	–
Primigravid	796 (60.9)	26.1	1.76 (1.39–2.23)	–	1.71 (1.32–2.20)	–
Gestational age				0.32		0.27
< 20 weeks	430 (32.9)	22.8	ref	–	ref	–
20–24 weeks	771 (58.9)	21.9	0.96 (0.77–1.20)	–	0.93 (0.75–1.16)	–
25–30 weeks	107 (8.2)	15.9	0.70 (0.44–1.11)	–	0.69 (0.43–1.08)	–
Socio-economic status§				< 0.001		< 0.001
Least poor	256 (19.6)	12.9	ref	–	ref	–
Less poor	252 (19.3)	12.3	0.95 (0.60–1.51)	–	0.87 (0.56–1.36)	–
Middle	257 (19.7)	22.6	1.75 (1.18–2.59)	–	1.41 (0.95–2.10)	–
More poor	259 (19.8)	26.6	2.07 (1.42–3.01)	–	1.62 (1.11–2.36)	–
Most poor	249 (19.0)	35.3	2.74 (1.91–3.93)	–	2.01 (1.39–2.90)	–
Bed net use previous night§				0.19		0.26
Yes	789 (60.3)	20.5	ref	–	ref	–
No	496 (37.9)	23.6	1.15 (0.93–1.42)	–	1.12 (0.92–1.37)	–

CI = confidence interval; PR = prevalence ratio; ref = reference.

* Crude PRs calculated from univariate Poisson regression.

† *P* values obtained from Wald’s test. Comparisons restricted to observations without missing data for variables listed.

‡ Adjusted PRs calculated using Poisson regression, adjusting for all other variables listed in the table.

§ Missing values for SES *N* = 35 (2.7%); bed net use *N* = 23 (1.8%).

**Table 4:** Distribution of risk factors, crude and adjusted prevalence ratios for malaria in pregnancy in The Gambia (crude *N* = 1,194; adjusted *N* = 1,113)

Variable	Frequency (%)	% *Plasmodium falciparum* infection	Crude PR* (95% CI)	*P* value†	Adjusted PR‡ (95% CI)	*P* value†
Months pregnant in rainy season				< 0.001		< 0.001
0 months	500 (41.9)	7.8	ref	–	ref	–
1 months	201 (16.8)	6.0	0.77 (0.41–1.43)	–	0.74 (0.39–1.40)	–
2 months	128 (10.7)	6.3	0.80 (0.38–1.67)	–	0.82 (0.37–1.86)	–
3 months	157 (13.2)	7.0	0.90 (0.47–1.71)	–	1.00 (0.54–1.84)	–
4 months	208 (17.4)	16.8	2.16 (1.41–3.31)	–	2.24 (1.45–3.47)	–
Age				< 0.001		0.05
25 years+	121 (10.1)	6.6	ref	–	ref	–
21–24 years	287 (24.0)	8.4	1.26 (0.58–2.74)	–	1.03 (0.49–2.19)	–
18–20 years	569 (47.7)	6.7	1.01 (0.48–2.11)	–	0.77 (0.38–1.59)	–
Under 18 years	217 (18.2)	16.1	2.44 (1.17–5.09)	–	1.52 (0.70–3.31)	–
Gravidity				0.03		0.06
Secundigravid	520 (43.5)	6.7	ref	–	ref	–
Primigravid	674 (56.5)	10.4	1.54 (1.05–2.28)	–	1.54 (0.98–2.43)	–
Gestational age				0.001		< 0.001
< 20 weeks	394 (33.0)	12.9	ref	–	ref	–
20–24 weeks	652 (54.6)	7.4	0.57 (0.39–0.83)	–	0.49 (0.33–0.71)	–
25–30 weeks	148 (12.4)	4.1	0.31 (0.14–0.71)	–	0.32 (0.14–0.73)	–
Socioeconomic status§				0.01		0.001
Least poor	228 (19.1)	4.4	ref	–	ref	–
Less poor	232 (19.4)	5.6	1.28 (0.57–2.86)	–	1.31 (0.59–2.91)	–
Middle	222 (18.6)	9.5	2.16 (1.04–4.48)	–	1.92 (0.93–3.98)	–
More poor	224 (18.8)	12.1	2.75 (1.36–5.55)	–	2.96 (1.45–6.04)	–
Most poor	211 (17.7)	12.8	2.92 (1.45–5.88)	–	3.36 (1.66–6.77)	–
Bed net use previous night§				0.61		0.36
Yes	976 (81.7)	8.7	ref	–	ref	–
No	182 (15.2)	9.9	1.14 (0.70–1.84)	–	1.25 (0.78–2.01)	–

CI = confidence interval; PR = prevalence ratio; ref = reference.

* Crude PRs calculated from univariate Poisson regression.

† *P* values obtained from Wald’s test. Comparisons restricted to observations without missing data for variables listed.

‡ Adjusted PRs calculated using Poisson regression, adjusting for all other variables listed in the table.

§ Missing values for SES *N* = 77 (6.4%); bed net use *N* = 36 (3.0%).

**Figure 2.**
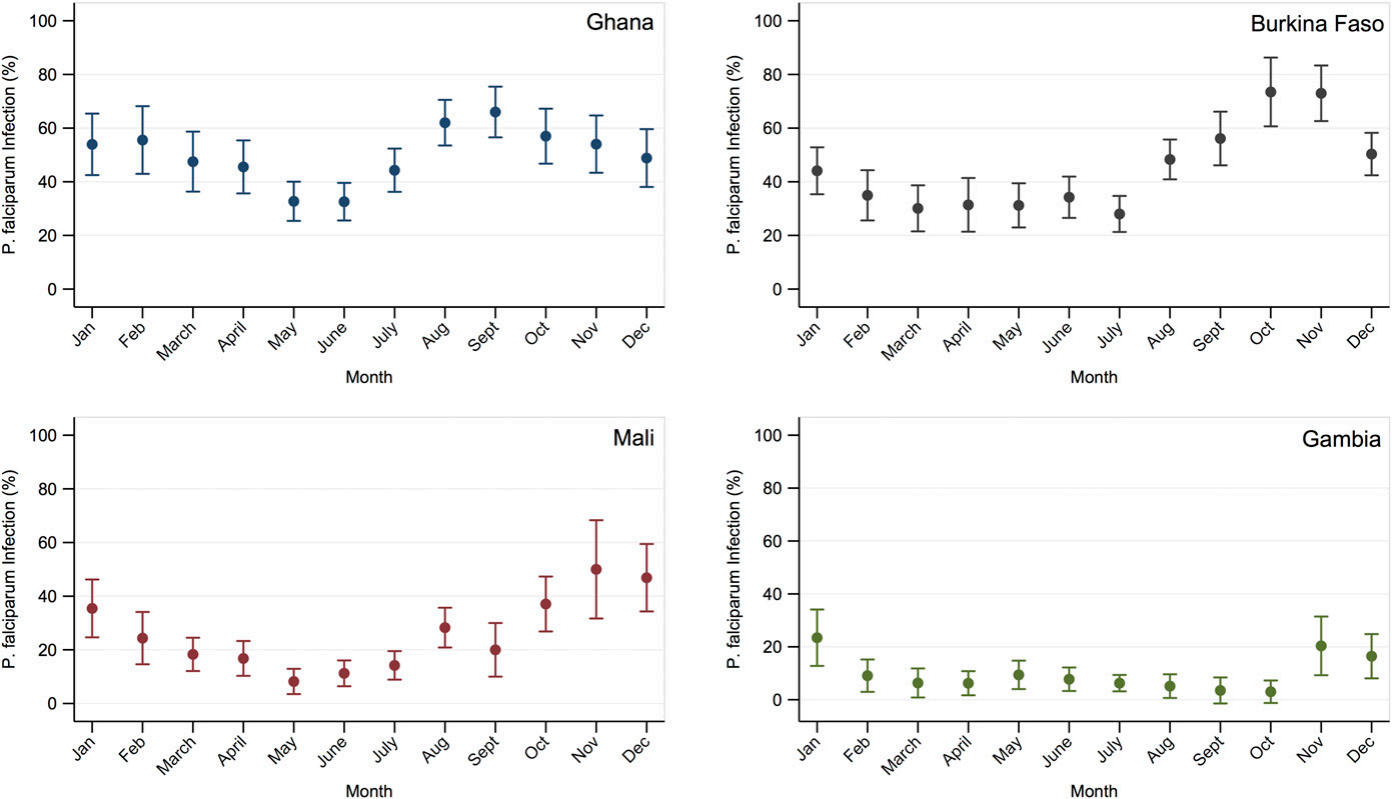
Prevalence of malaria caused by *Plasmodium falciparum* infection at first ANC visit by month in Ghana, Burkina Faso, Mali, and The Gambia. This figure appears in color at www.ajtmh.org.

**Figure 3.**
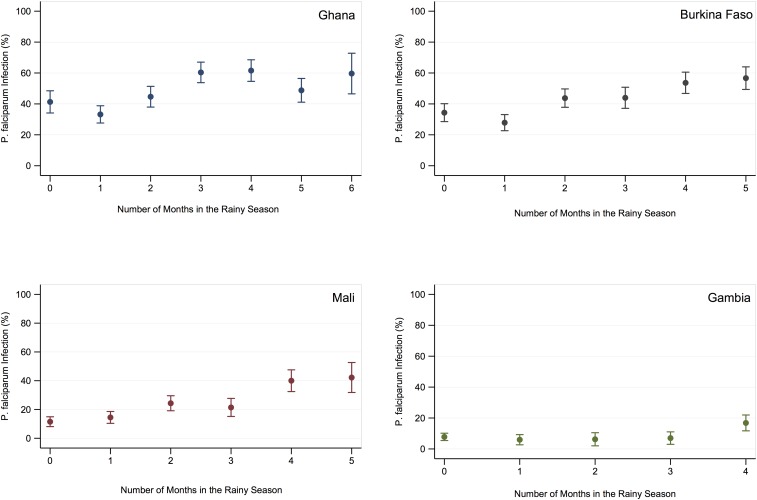
Prevalence of malaria caused by *Plasmodium falciparum* infection at first ANC visit by duration of pregnancy in the rainy season in Ghana, Burkina Faso, Mali, and The Gambia. This figure appears in color at www.ajtmh.org.

The overall prevalence of malaria at first ANC visit was 48.0% (95% CI: 45.2–50.7) in Ghana, 42.0% (95% CI: 39.4–44.5) in Burkina Faso, 21.7% (95% CI: 19.5–23.9) in Mali, and 8.8% (95% CI: 7.2–10.4) in The Gambia. When estimated by month of ANC attendance, prevalence was greatest in the late wet season and early dry season months in each country (Figure 2). In all four countries, prevalence increased with greater duration of pregnancy in the rainy season, among those having the longest exposure prevalence was 59.7% (95% CI: 46.5–72.8), 56.7% (95% CI: 49.4–64.0), 42.2% (95% CI: 31.8–52.6), and 16.8% (95% CI: 11.7–22.0), respectively (Figure 3). Prevalence was still substantial among women whose entire pregnancy before first ANC visit had occurred in the dry season (i.e., 0 months in high transmission season): 41.3% (95% CI: 34.1–48.5) in Ghana, 34.4% (95% CI: 28.6–40.1) in Burkina Faso, 11.5% (95% CI: 8.1–14.9) in Mali, and 7.8% (95% CI: 5.4–10.2) in The Gambia (Tables 1–4).

### Risk factors for MiP.

In crude analyses, there were very strong associations in all countries between malaria infection on presentation at ANC and the duration of pregnancy spent in the rainy season, as well as age and gravidity. Women whose entire pregnancy up to their first presentation at ANC had taken place during the rainy season were at greater risk of malaria than those whose entire pregnancy had taken place during the dry season (Ghana PR: 1.44, 95% CI: 1.10–1.90, *P* < 0.001; Burkina PR: 1.65, 95% CI: 1.34–2.04, *P* < 0.001; Mali PR: 3.67, 95% CI: 2.51–5.38, *P* < 0.001; Gambia PR: 2.16, 95% CI: 1.41–3.31, *P* < 0.001). The prevalence of infection at first ANC visit was also greatest in younger mothers (decreasing with increasing age), and was greater among primigravid women as compared with secundigravid women. In addition, there was strong evidence of an association between lower SES and greater prevalence of infection in Ghana, Mali, and The Gambia; however, this was not seen in Burkina Faso (Tables 1–4). Women presenting to ANC at later gestational ages were at lower risk of malaria than those presenting earlier, and bed net use was not strongly associated with malaria prevalence in crude analyses.

After adjusting for each of these factors (i.e., age, gravidity, gestational age, SES, net use, and duration of pregnancy in the rainy season) in multivariable models, the association between malaria and duration of pregnancy in the rainy season remained very strong in all four countries. The prevalence of infection among those whose pregnancy spanned the entire rainy season was 1.48 (95% CI: 1.13–1.95, *P* < 0.001) times that of the prevalence among those whose pregnancy spanned the dry season in Ghana, 1.85 (95% CI: 1.50–2.29, *P* < 0.001) times in Burkina Faso, 3.16 (95% CI: 2.12–4.70, *P* < 0.001) times in Mali, and 2.24 (95% CI: 1.45–3.47, *P* < 0.001) times in The Gambia.

After adjustment, other well-known risk factors for MiP, including young maternal age and primigravidity, remained associated with infection (Tables 1–4). We observed a lower prevalence of infection among nonbed net users in Ghana (PR compared with net users: 0.85, 95% CI: 0.76–0.96, *P* = 0.01) (Table 1), but no association between net use and infection in Burkina Faso, Mali, and The Gambia after adjustment (Tables 2–4).

## DISCUSSION

In areas of seasonal malaria transmission in Ghana, Burkina Faso, Mali, and The Gambia, the prevalence of malaria infection at the time of first ANC contact in pregnancy was generally high, with two countries having prevalence of infection more than 40% by microscopy. The risk of infection was found to increase markedly as the number of months of pregnancy that had occurred in the rainy season before attending ANC increased, with women whose pregnancies spanned most of the rainy season at the highest risk.

Given the well-known seasonal fluctuations in infection risk in West Africa,^17–19^ increasing risk with increasing time spent exposed during the rainy season is not unexpected. However, an important finding, which has been much less well described, was that women whose pregnancy until first ANC contact had occurred largely—or entirely—in the dry season were still at substantial risk of carrying infections in pregnancy. This was particularly the case in the two high transmission countries studied, Ghana and Burkina Faso, where women who carried none of their pregnancy in the wet season up to their first ANC visit still had a malaria prevalence of 30–40%. New infections are unlikely to explain such a high prevalence, given the low risk of infection in the months leading up to these women attending ANC. Consequently, it is likely that the majority of these infections were acquired before pregnancy in a previous transmission season, and retained until, and also during, the woman’s current pregnancy. A previous modeling study by Walker et al.^11^ suggested that in all but the very highest transmission settings, more than 50% of the time spent infected in pregnancy results from infections acquired before development of the placenta, with the majority of these infections acquired pre-pregnancy. The impact of these retained infections, which is mirrored in all four countries with different seasonal transmission patterns, has not yet been well described. However, there is increasing evidence of the deleterious effects of malaria in the first trimester, including its impact on development of the placenta.^27^ This impact may be substantial, given both the high prevalence of infection and the likelihood that if these infections were acquired before pregnancy, they have lasted for several months, thus spanning the entire first trimester and part of the second trimester.

In all four countries, the risk of malaria was also found to be greatest among women in their first pregnancy, younger women, those presenting at an earlier gestational age, as well as those of lower SES, with these factors remaining associated with infection in multivariable analyses. These factors are consistent with results found in other seasonal,^18,28,29^ as well as nonseasonal, settings such as Mozambique^30^ and Gabon.^17^ We also found that net use was not associated with prevalence, except in Ghana where nonusers had lower prevalence (potentially because current net use may be a marker of living in areas with higher biting rates, and consequently higher malaria risk).

Currently, the World Health Organization (WHO) recommends universal bed net coverage to prevent malaria across sub-Saharan Africa, but the usage has consistently been found to be lowest in adolescents and young adults.^14,31^ The WHO’s pregnancy-specific recommendations include 1) effective case management of clinical malaria, 2) IPTp with SP at each ANC contact after the first trimester of pregnancy, and 3) provision of insecticide-treated bed nets at ANC.^9^ However, the women most likely to be infected, and those most likely to suffer negative consequences of MiP—young, primigravid women—will likely not benefit from these strategies until they first contact ANC.^13,14^ The observed high prevalence suggests that relatively few women develop clinical malaria and receive effective treatment. Infected women will benefit from having their infections cleared by IPTp with SP, but the full benefit of chemoprevention may not be realized if pregnant women have already spent the first part of their pregnancy—perhaps more than half of the total gestation period—infected. Likewise, bed nets delivered at first ANC visit will provide protection for the remainder of a woman’s current pregnancy and in subsequent pregnancies if nets are retained.^15,32^ However, this leaves primigravid women, who are already at higher risk, unprotected until first ANC. This was reflected within our dataset, with higher net use during second pregnancies in all four countries.

Our results therefore emphasize the need for enhanced and effective prevention either at an earlier stage of pregnancy or before pregnancy—perhaps by measures targeting all women of child-bearing age or young women and adolescent girls. For instance, pre-pregnancy interventions could include net delivery campaigns targeting this population (i.e., young women or all women of child-bearing age), presumptive treatment, or perhaps rapid diagnostic test (RDT)–based screening aiming to reduce the infectious reservoir in young women. Seasonally targeted MiP interventions during early pregnancy have also been suggested as a way to meet the different needs of women in seasonal settings. These could include enhanced IPTp, seasonal drives to increase net coverage, and potentially even seasonal vaccination of pregnant women with RTS,S/AS01, provided the vaccine is found to be nonteratogonic.^33^

Although our study evaluates the prevalence of, and risk factors for, MiP in four West African countries, several limitations of the design should be noted. First, it is not necessarily the case that there is no malaria transmission during the dry season, particularly in areas where prevalence is higher, and thus where residual transmission in the dry season may be greater. Consequently, a small percentage of infections seen in women only pregnant during the dry season may result from recent infection. However, we consider it unlikely that the high prevalence observed (particularly in Ghana and Burkina Faso) can result only from recent infections. Although out of the scope of our analysis, this could be further examined empirically in a prospective study, perhaps by following women from marriage into first pregnancy and typing their parasites by molecular markers. Secondly, our study design is a cross-sectional analysis of baseline data of women enrolled in a multicenter trial. Primigravid or secundigravid women attending ANC for the first time were enrolled with relatively few exclusion criteria (i.e., a severe chronic infection, known allergy to SP, prior receipt of SP during their pregnancy, or an intention to leave the study area), and with less than 200 refusals among more than 6,500 women screened.^12^ However, this is not a truly random sample of all ANC attendees. Given the findings in relation to SES in this study, and more generally, we speculate that prevalence may be even greater if we could include women unable to attend ANC, or any women who suffered a miscarriage as a result of their infection early in pregnancy and who consequently never attended ANC.

In this study, bed net use was defined as whether women reported sleeping under a net the night before their first ANC visit. A possible limitation of our study is that, although this question is widely used and validated, net use at the time of first ANC contact does not necessarily represent the net use at the etiologically relevant time (when the women actually became infected). In addition, we recognize that our measures of gestational age by symphysis-fundal height may not be as robust as methods such as ultrasound, which were not available in these settings. However, given that women had repeated contact with ANC until delivery, the gestational age estimates were crosschecked with measurements taken at the later contacts, and any woman with inconsistent estimates of gestational age were set to missing and therefore not included in our analysis.^12^ Furthermore, our results, based on microscopy, may be somewhat conservative in terms of the overall burden that might have been detected by molecular methods. A study by Williams et al.^34^ which analyzed samples from half the women enrolled in this study using polymerase chain reaction, indicated that although the sensitivity of microscopy and RDT were high in paucigravid women, additional lower density infections were still missed. However, these additional low-density infections may be of less concern with respect to outcomes of pregnancy.^35^

## CONCLUSIONS

We found a substantial burden of malaria infection at the time of first contact with ANC among primigravid and secundigravid women in West Africa. Although there is evidence of increasing prevalence among women whose pregnancy occurs during the rainy season, even women pregnant at a time of low immediate risk had high rates of parasite carriage, likely due to infections acquired before their pregnancy. Additional strategies are needed to prevent MiP, but for maximum impact, these will need to address infections acquired before pregnancy and carried during the first months of pregnancy before contact with ANC.

## Supplementary Material

Supplemental Figure and Table.
