# Supplementary material for: Seasonal Dynamics of Malaria in Pregnancy in West Africa: Evidence for Carriage of Infections Acquired Before Pregnancy Until First Contact with Antenatal Care
Source: Am J Trop Med Hyg. 2017 Dec 4;98(2):534–42. doi: 10.4269/ajtmh.17-0620 (PMC5929207; doi:10.4269/ajtmh.17-0620)
Supplement: Supplementary file 1 [file tpmd170620.SD1.pdf]

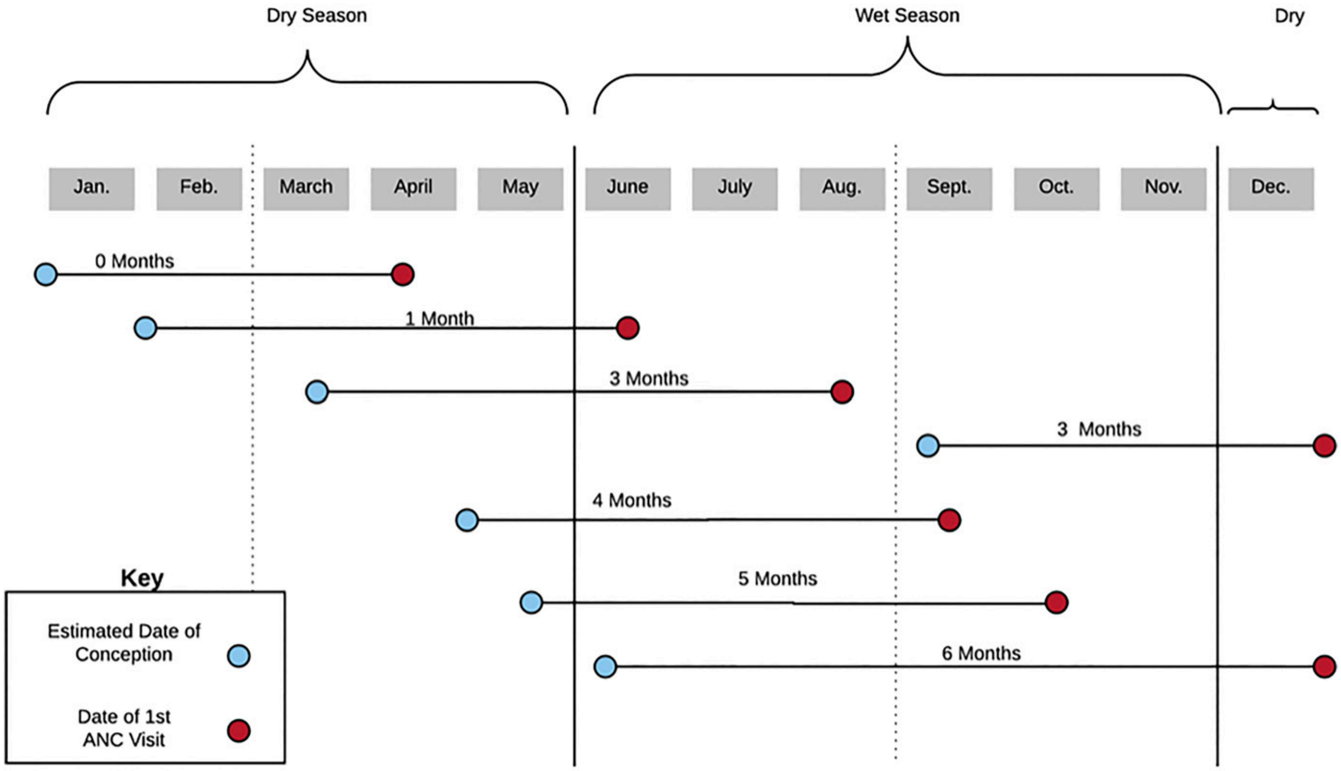

Supplemental Figure 1. Illustration of the duration of pregnancy in the rainy season measure in Ghana. NB: Estimated date of conception calculated from gestational age measure in weeks (measured by symphysis-fundal height).

SUPPLEMENTAL TEXT 1

Household assets and amenities used to create socioeconomic status quintiles. The wealth index was based on the following durable household assets and amenities: drinking water source; type of toilet facility; cooking fuel; type of flooring; type of roofing; household electrification; and ownership of a radio, television, fixed telephone, refrigerator, mobile phone, bicycle, scooter, car, ox-cart, and animals (chicken, ducks, goats).

SUPPLEMENTAL TEXT 2

Ethical approval committees.

Country	Ethics committee
Burkina Faso	Comité national d’ethique pour la recherché en santé (CNERS) Ministère de la Santé, BP 7009, Ouagadougou 03, Burkina Faso
The Gambia	The Gambia Government/MRC laboratories Joint Ethics Committee, C/O MRC Laboratories, Fajara, P. O. Box 273 Banjul, The Gambia
Ghana	Ethics committee of the Ghana Health Service Committee Research & Development Division, Ghana Health Science, P. O. Box 190, Accra, Ghana
	Navrongo Health Research Center Institutional Review Board, Navrongo Health Research Center, P. O. Box 114, Navrongo, Ghana
Mali	Ethics committee of the Ministere de l’Enseignement Superieur et de la Recherche Scientifique Universite de Bamako-BP 1805- Bamako, Mali

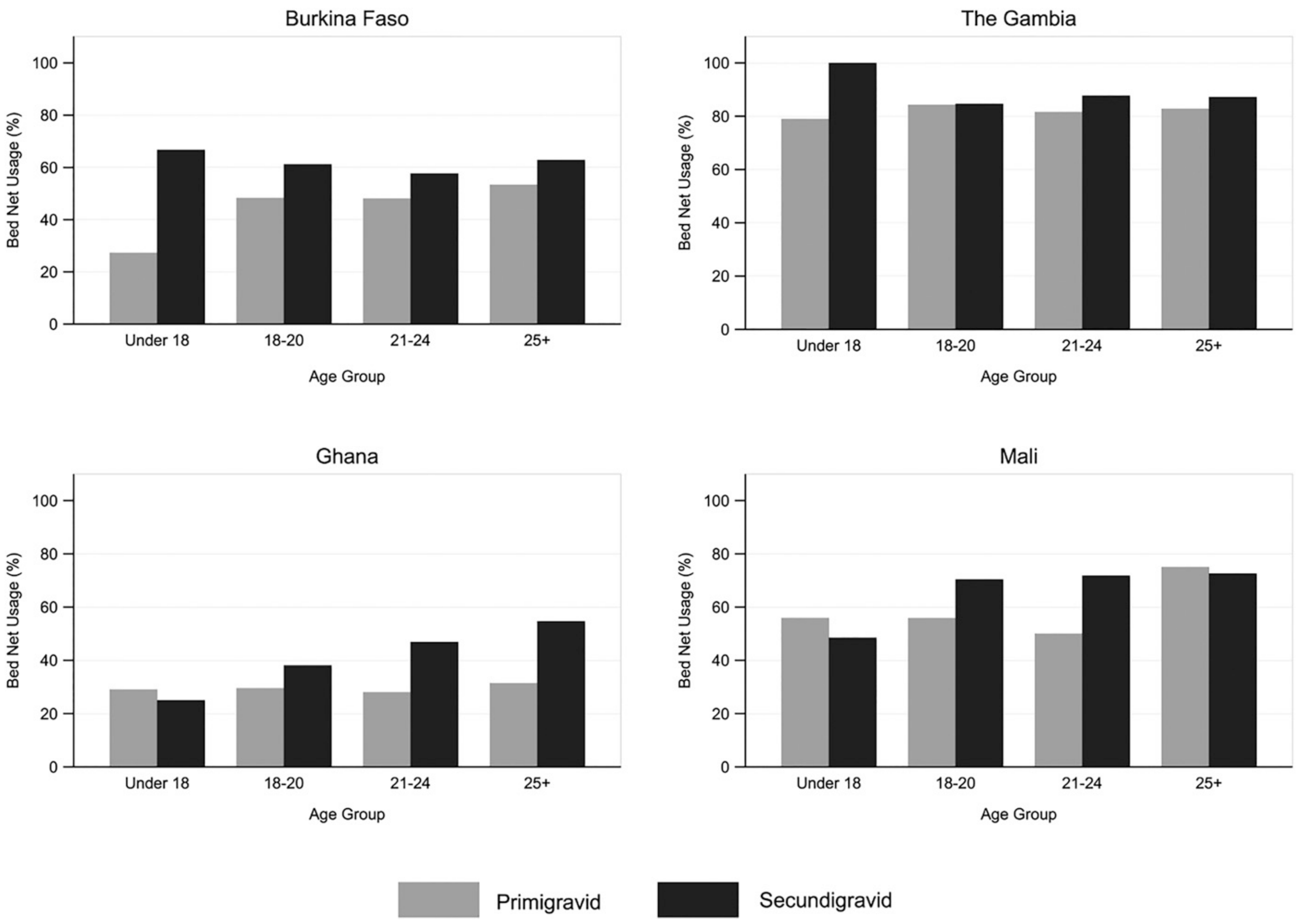

Supplemental Figure 2. Prevalence of bed net use at first ANC visit by age and gravidity in Burkina Faso, The Gambia, Ghana, and Mali.
